# Obituary

**Published:** 1850-05

**Authors:** 


### ARTICLE IX.

### OBITUARY.

We regret to learn by the newspapers, the death of JOHN H. SANDERS, M.D. of *Indianapolis* Ia, who was on his way from New Orleans to his home, when stricken down by the Cholera. He was one of the oldest members of our profession in Indiana. He spent a long series of years in the faithful discharge of his professional duties in Indianapolis; where he lived highly respected; and his loss is deeply regretted.

It has seldom been our good fortune to enjoy the friendship of one so richly endowed with those kindly feelings, and impulses of the heart, that form the true gentleman, and ornament the christian. E.

We have learned through the public prints, the death of our friend, Dr. T. W COWGILL of Greencastle, Indiana. He was selected to fill the chair of the Principles and Practice of Medicine in the Indiana Central Medical College; but his declining health prevented him from entering on the duties assigned him. Dr. C. was a popular and useful citizen in the community where he resided, and leaves a wide circle of relatives and friends to deplore his loss. His disease was Phthisis. M.

Died, in Boston, on the 2d of March last, Dr. JNO. D. FISHER, one of the Physicians to the Massachusetts General Hospital; aged 53 years.

Died, at Ottawa, Sept. 17th. 1849, Dr. HENRY S. PRATT. The deceased was a young physician of great promise, evincing, as he did, an unusual facility in acquiring, and a great fondness for the practice of, his profession. Dr. Howland, (his preceptor,) made some feeling remarks before the Ottowa Medical Society, on the occasion of Dr. Pratt's death; and we designed publishing them, but find we have not the space.

